# 

**Published:** 1919-06-21

**Authors:** 


### MATRONS' AND SISTERS' APPOINTMENTS.

AMERICAN RED CROSS MATERNITY HOSTEL, GRANGE ROAD, BERMONDSEY.—Miss J. L. Holmes and Miss L. J. Wills have been appointed matron and assistant matron respectively. Miss Holmes was trained at Kensington Infirmary, has been unit administrator in the Q.M.A.A.C., and has held various appointments as matron and superintendent in hospitals in England and France. Miss Wills was trained at the West Derby Union Infirmary, where she was later staff nurse and sister, and has been ward sister at the Manor War Hospital.

BIRMINGHAM HOSPITALS FOR DISABLED MEN.—Miss E. E. Fletcher, R.R.C., has been appointed matron. There were seventy-eight applications for the position. Miss Fletcher was trained at the Royal Albert Edward Infirmary, Wigan, where she was later ward sister and assistant matron. Since the commencement of the war Miss Fletcher has been matron of the 2nd Western General Hospital, Manchester.

BIGNOLD COTTAGE HOSPITAL, WICK.—Miss Jessie A. Aitken has been appointed matron. She was trained at Aberdeen Royal Infirmary, and has worked as a Queen's nurse at Edinburgh, Clydebank, and Wick. She has also been staff nurse and sister in military hospitals in Aberdeen and France.

CRIPPLES' HOME, STOKE-ON-TRENT.—Miss A. E. Taylor, A.R.R.C., has been appointed matron. She was trained at St. Bartholomew's Hospital, E.C., and was later sister at St. Bartholomew's Hospital, Rochester; assistant matron of the Adelaide Hospital, Dublin; matron of a hostel for girls at Woolwich, and has done temporary duty at the Royal Free Hospital, Gray's Inn Road.

HASTINGS, MINISTRY OF PENSIONS HOSPITAL, "OLD HASTINGS HOUSE."—Miss Waller-Senior has been appointed matron. She was trained at the Madras General Hospital, India, and left India soon after the outbreak of war. She was later sister at the Tooting Military Hospital, and sister-in-charge of "Old Hastings House" Red Cross Hospital.

HOSPITAL FOR CRIPPLED CHILDREN, LEASOWE.—Miss Mary Keeley and Miss Ethel Baynes have been appointed night sister and housekeeping sister respectively. Miss Keeley was trained at the Royal Infirmary, Manchester, and has since been doing military nursing. Miss Baynes was trained at King's College Hospital, and has been sister at the private patients' block at the South London Hospital for Women.

MENTAL HOSPITAL, RYHOPE, SUNDERLAND.—Miss K. Johnson has been appointed assistant matron. She was trained at Manchester Royal Infirmary and Larbert Mental Hospital. She has been staff nurse at the former institution, and at the Second Western General Hospital, Manchester.

METROPOLITAN BOROUGH OF HAMMERSMITH MATERNITY HOSPITAL.—Miss Edington has been appointed matron. She was trained at Mile End Infirmary and Queen Victoria's Institute, Northampton, has been county superintendent, Cambridgeshire, and matron of the Norwich Maternity Institution, and of St. Mary's Hostel, Croydon.

MIDDLETON-IN-WHARFEDALE SANATORIUM.—Miss E. W. Taylor has been appointed matron. She was trained at St. Bartholomew's Hospital, and has since been assistant matron of the Adelaide Hospital, Dublin; matron of the Royal National Hospital for Consumption, Newcastle, Co. Wicklow, of the Dublin Red Cross Hospital, and of Moxley Sanatorium, Wednesbury. She has also seen military service at Malta.

ROYAL EDINBURGH ASYLUM.—Miss Margaret Greigor, Miss Catherine Lang McPherson, and Miss Margaret Keith have been appointed assistant matrons. Miss Greigor was trained at the Royal Edinburgh Asylum and the Royal Victoria Infirmary, Newcastle-on-Tyne; Miss McPherson was trained at Aberdeen Royal Infirmary and at Lambeth Infirmary; Miss Keith at Fife and Kinross Asylum and at the Eastern Hospital, Dundee. She has also had fever experience at the King's Cross Hospital, Dundee.

ROYAL BUCKS HOSPITAL, AYLESBURY.—Miss A. Whittleton has been appointed sister. She was trained at Salisbury General Infirmary, and has since been staff nurse at the National Hospital, Queen Square, and sister at the Miller General Hospital, Greenwich. She has had fever and massage experience.

ROYAL VICTORIA AND WEST HANTS HOSPITAL, BOSCOMBE.—Miss Dorothy Parry has been appointed assistant matron. She was trained at the Royal Southern Hospital, Liverpool, where she was later sister, has been for two and a-half years at the Liverpool Merchants' Mobile Hospital in France, and night sister at the Children's Hospital, Leasowe, Cheshire.

TETBURY COTTAGE HOSPITAL, GLOUCESTERSHIRE.—Miss E. E. Southgate has been appointed nurse-matron. She was trained at St. Thomas's Hospital, has been sister at the Throat and Ear Hospital, Brighton, the Cottage Hospital, Walton-on-Thames, and at the 3rd Southern General Hospital, Oxford, and nurse-matron of the Shedfield Cottage Hospital, Hants.

WARNEFORD HOSPITAL, LEAMINGTON.—Miss Hilda Ellerker has been appointed sister. She was trained at Queen Mary's Hospital for the East End, Stratford, has been ward and theatre sister at Paddington Green Children's Hospital, and ward sister at the Princess Club Hospital, Jamaica Road, Bermondsey.

HAYLING ISLAND, LORD MAYOR TRELOAR'S HOSPITAL, SANDY POINT BRANCH.—Miss Brownley has been appointed sister-in-charge. She was trained at the Victoria Children's Hospital, Hull, and at University College Hospital, where she was later ward and night sister. She has since been assistant matron of Sheffield Royal Infirmary, matron of Birmingham Eye Hospital, nursing sister under Queen Alexandra's Imperial Military Nursing Service Reserve, and assistant matron of Wootton Auxiliary Hospital, Liverpool. Miss Davies has been appointed ward sister. She was trained at the Belgrave Hospital for Children and at University College Hospital, where she was later on the private staff. She has also been night sister at the Royal Waterloo Hospital for Women and Children, ward sister at the Norfolk and Norwich Hospital, holiday sister at University College Hospital, and nursing sister under Queen Alexandra's Imperial Military Nursing Service Reserve.

ALTON, LORD MAYOR TRELOAR'S HOSPITAL, SPECIAL TREATMENT BLOCK.—Miss Wilkinson has been appointed sister-dispenser. She was trained at St. Bartholomew's Hospital, E.C., where she was later night superintendent, ward and out-patients' sister, and has since been sister at Pinewood Sanatorium, Wokingham, matron of Matlock Sanatorium, Matlock, temporary matron and night sister at Ramsgate General Hospital, and temporary night sister at the Royal Sea Bathing Hospital, Margate. Miss Mace and Miss Latham have been appointed ward sisters. Miss Mace was trained at Cheltenham General Hospital, and has since been sister at the Royal Hospital for Sick Children, Edinburgh. Miss Latham was trained at Hereford General Hospital, and has since been night and home sister at Shoreditch Infirmary and night superintendent at Mile End Military Hospital.

ISOLATION HOSPITAL, NORWICH.—Miss Florence Tilt has been appointed ward sister. She was trained at the City Hospital, Little Bromwich, Birmingham, and at Worcester General Hospital.

OCKENDEN CONVALESCENT HOME, TORQUAY.—Miss Rose M. Hutty has been appointed lady superintendent. Miss Hutty was trained at the Royal Devon and Exeter Hospital, Exeter, and was appointed sister of the Summerhayes ward. On the outbreak of war she was appointed to the post of matron, No. 2 Section, Military Hospital, Exeter.

TYNEMOUTH UNION HOSPITAL, NORTH SHIELDS.—Miss Martha Agnes Rodgers has been appointed home sister. She was trained at Birmingham Union Infirmary, and has been ward sister and assistant matron at the Norfolk War Hospital.

### THE COLLEGE OF NURSING (Limited by Guarantee).

NOTTINGHAM CENTRE.

*Friday, June 27.*—Lady Markham is receiving the members of this Centre to Newstead Abbey to see the Abbey and the grounds. All nurses who are able to avail themselves of this invitation should send their name to Miss Smith, 4 Park Terrace, Nottingham, who is acting as Secretary during the absence of Miss Taylor on holiday.

LONDON CENTRE.

THURSDAY, July 3 (3 P.M.), and THURSDAY, July 24 (11 A.M.). —Messrs. Edward Cook and Sons, soap specialists, of Bow, are kindly conducting fifteen nurses over their factory. Any members wishing to join the party on either day are requested to communicate with the Secretary of the London Centre, Miss Biggar, St. Thomas's Hospital, S.E.

### Buildings Contemplated and in Progress.

ANGLESEA.—The Anglesea County Council have asked the British Red Cross Society to establish an isolation hospital in the county.

BAKEWELL.—Funds are being raised for building a cottage hospital, estimated to cost £10,000.

BANBURY.—It has been decided to enlarge the local infirmary at an estimated cost of £25,000.

BERMONDSEY.—The Borough Council has recommended the local War Memorial Committee to provide a children's hospital for Bermondsey.

BRADFORD.—The Corporation Health Committee propose to lease from the Guardians the St. Luke's War Hospital, when it is vacated by the Army Council, and to adapt the premises as a public general hospital for the city.

BRANDON.—The Urban District Council have decided to extend the isolation hospital.

CASTLE-AN-DINAS.—The Cornwall Sanitary Committee have approved a scheme for adapting the Castle-an-Dinas property as a hospital for venereal diseases at a cost of £3,500.

CLIVEDEN.—It is proposed to enlarge and improve the Berks and Bucks Joint Sanatorium at Cliveden, at a cost of £19,000.

CREWE.—It is proposed to make additions to the Cottage Hospital at a cost of £2,000.

CROOK.—It has been decided to build a permanent hospital at Etherley, Crook, as soon as sufficient funds are subscribed.

GALASHIELS.—The Town Council has chosen a site for a hospital for infectious diseases.

GRIMSBY.—Plans are being prepared for an extension of the administrative block at the Scartho Sanatorium.

HALTWHISTLE.—A sum of £3,000 has been raised for a new hospital.

HEANOR.—A gift of a site from Mr. Miller Mundy has been accepted by the War Memorial Committee for the new hospital.

HENLEY-ON-THAMES.—It has been decided to build a cottage hospital as a war memorial. The estimated outlay will be from £15,000 to £20,000.

HEXHAM.—It has been decided to build a cottage hospital as a war memorial.

HOLMFORTH.—It is proposed to establish a hospital at Holmforth as a peace memorial. The local committee has purchased the Elmwood Estate, and about £7,000 has been promised towards the cost.

HORSFORTH.—The local war memorial is to take the form of a cottage hospital.

HULL.—At the last meeting of the Corporation Health Committee authority was given for taking out quantities and advertising for tenders for a new infectious diseases hospital.

LONDON, E.—Extensions of the City of London Hospital for Diseases of the Chest have been decided upon.

MALTON.—As a war memorial it has been decided to appeal for £10,000 for a new hospital for the district.

MANCHESTER.—It is proposed to build a children's hospital at an estimated cost of £25,000.

SUTTON.—The war memorial is proposed to take the form of a fifty-bed hospital.

